# Ensuring a sustainable supply of aquatic products in the future: research on the promotion and application of deep-sea aquaculture

**DOI:** 10.3389/fnut.2025.1643753

**Published:** 2025-08-20

**Authors:** Ye Ma, Ying Zhang

**Affiliations:** ^1^School of Management, Ocean University of China, Qingdao, China; ^2^School of Management, Ocean University of China, and the Institute of Marine Development, Ocean University of China, Qingdao, China

**Keywords:** deep-sea aquaculture, aquatic product supply, evolutionary game model, government intervention, market mechanism

## Abstract

Fisheries are an important source of protein for humans. Currently, freshwater and coastal aquaculture fisheries, as well as capture fisheries, have reached saturation point in terms of development potential and are severely polluted, making the supply of aquatic products unsustainable. Deep-sea aquaculture utilizes the vast exploitable space of the open sea, breaking through the limitations of coastal waters and land. It directly increases the global supply of high-protein aquatic products through large-scale and green aquaculture, playing an important role in ensuring the sustainable supply of aquatic products in the future and meeting human nutritional needs. Therefore, based on the assumptions of bounded rationality and stakeholder interdependence, this study constructed an evolutionary game model to analyze how to promote the application and implementation of deep-sea aquaculture. The conclusions of the study are as follows. First, this study affirms that the government plays an important role in promoting and applying deep-sea aquaculture, but government intervention cannot be sustained. Second, blind government intervention is not beneficial and may lead to a double failure of government and market. Finally, compared to the expected total profits from deep-sea aquaculture, it is more important to identify risk and cost thresholds and establish a fair mechanism for sharing benefits and risks. This threshold is defined as the total risk and costs should be less than the sum of potential profits and government subsidies. The research conclusions indicate that the government’s role in the promotion and application of deep-sea aquaculture should evolve in tandem with the industry’s maturity, transitioning from a “leading role in the initial phase” to a “guiding role in the mature phase.” Additionally, the fundamental approach to promoting the widespread adoption of deep-sea aquaculture lies in establishing a cooperative mechanism between aquaculture enterprise and service organization under the guidance of market mechanisms. This study aims to explore how to promote deep-sea aquaculture to enhance the sustainable supply of global aquatic products, thereby expanding and securing channels for human food and protein supply.

## Graphical abstract

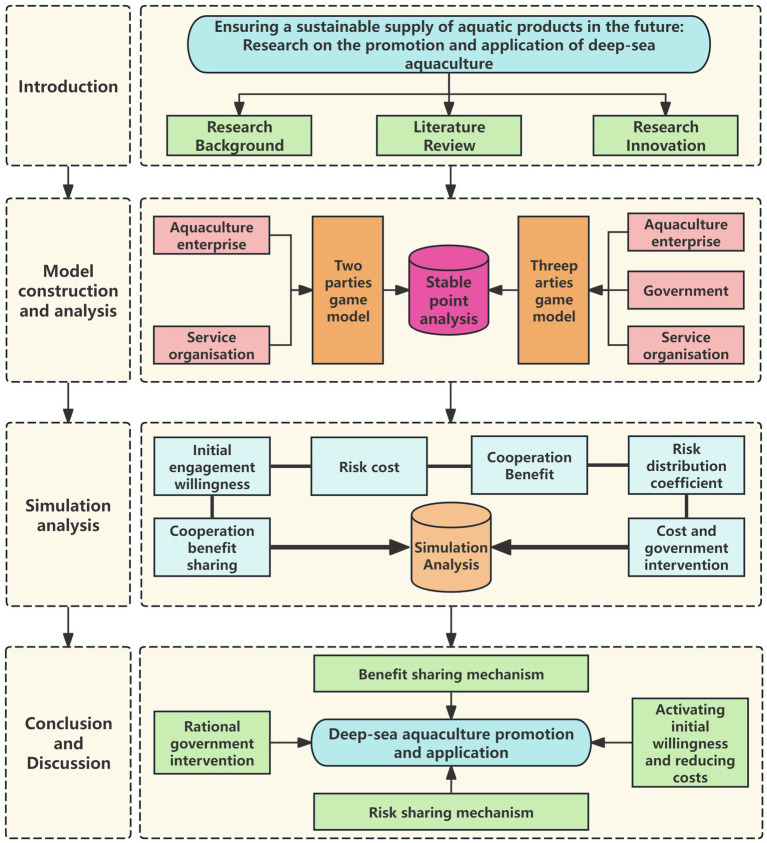

## 1 Introduction

Deep-sea aquaculture refers to aquaculture activities conducted in offshore marine areas that are farther from the coast, have deeper water bodies, and more open environments. Currently, there is no unified or clear definition of deep-sea aquaculture. The Food and Agriculture Organization of the United Nations (FAO) defines Deep-sea aquaculture as an offshore production system that is set up in open sea areas exposed to wind and wave action, secured by facilities and equipment, and supported by supply vessels (1). The United States defines deep-sea aquaculture as a fishery farming industry outside the 12 nautical mile offshore and territorial sea, but within the country’s Exclusive Economic Zone, while Europe defines deep-sea aquaculture as a fishery farming industry that conducts its production activities in the high seas area (2). In China, it is defined as a large-scale deep-sea aquaculture method with large-scale fishery equipment as the main tool, supported by mechanization, intelligence, automation and other technologies (3).

As the global population grows and living standards improve, the demand for high-quality animal proteins such as aquatic product continues to rise. By 2050, the global population is projected to exceed 9 billion people. Under current consumption levels, the demand for protein is expected to increase by over 20% (4). Fisheries play a crucial role in meeting human protein needs. According to statistics, the global fisheries production in 2020 reached 178 million tons, accounting for 17.5% of total protein supply (5). However, freshwater and coastal aquaculture capacity is now reaching saturation point, with extensive farming methods, excessive stocking densities, and overfishing leading to a sharp decline in natural fishery resources. Marine fishery ecological efficiency remains at a low level (6), posing challenges to the sustainability of the aquatic product supply system. Therefore, it is difficult for traditional fisheries to continue to meet the large aquatic consumption of the world’s population without sustainable transformation (7). Deep-sea aquaculture, on the one hand, can effectively increase the supply of aquatic product without occupying additional land resources, while utilizing more advanced aquaculture equipment and exerting less environmental pressure. It is currently the primary direction for the development of marine aquaculture (8–10). On the other hand, developing deep-sea aquaculture can effectively promote the concept of a “big food vision,” utilizing the superior water quality of distant sea areas to produce nutrient-rich marine fisheries (e.g., salmon), thereby expanding food supply channels and high-quality animal protein sources (11–13). Based on this, this study aims to provide theoretical support and practical guidance for the promotion and application of deep-sea aquaculture from a game theory perspective by constructing a stakeholder game analysis model. By promoting the development and application of deep-sea aquaculture, this study further aims to ensure the sustainable supply of aquatic product.

Research on deep-sea aquaculture has focussed firstly on top level design type studies. Based on the urgency of the current fishery transformation and the abundant advantages of deep-sea aquaculture, governments have made great efforts to promote the development of deep-sea aquaculture. The United States and Norway are the pioneer countries in the world in promoting the application of deep-sea aquaculture, and the deep-sea aquaculture technology of Atlantic salmon in Norway is very mature at present (14, 15), which mainly stems from the fact that the government has provided sufficient policy support for deep-sea aquaculture in terms of funding, technology, and industrial development (16, 17). In China, support for deep-sea aquaculture has been rising since 2011, and the number of relevant policies launched has been increasing, however, problems such as low efficiency of relevant policies, lack of articulation between policies, imperfect relevant supporting facilities, unbalanced policy support, and enterprise support to be improved have also been exposed (18–20). Based on the existing problems, scholars have proposed measures to strengthen planning guidance (21), enhance demand-based policy formulation and promotion (22), promote the establishment of demonstration zones (23), and optimize the farming space (24) from the perspective of top level design. This study summarizes the stakeholders covered in the above literature, as shown in Table 1. In addition to the policy top level design type of research, the current academic research on deep-sea aquaculture is also mainly focused on aquaculture equipment and technology analysis. Specifically, these can be classified as: research on hydrodynamic characteristics of aquaculture nets and workboats (25, 26); improvement and optimization of aquaculture nets, aquaculture workboats, and aquaculture platforms (27–31); selection of aquaculture methods and equipments for different fish species (32–34); innovation of automated fish detection technology (35); research on the improvement of net box feed feeding method and automatic feeding technology (36, 37).

**Table 1:** Coverage of stakeholders in existing literature.

Stakeholder perspective	Source of literature
Government	(16–19, 21, 23)
Aquaculture enterprise	(20, 22, 24)

In summary, the existing literature on deep-sea aquaculture has laid a solid theoretical foundation for this study. However, at present, the mechanism for the promotion and application of deep-sea aquaculture has not yet been established, and the existing researches are mainly in the categories of engineering technology and top level design, which are insufficiently researched as follows: (1) Deep-sea aquaculture not only needs to consider government support and technology development, but also needs to consider the complex game of interests between aquaculture enterprise, service organization and government. However, the research mainly focuses on a single participant and a single factor as the object of research, and lacks of research and consideration of other stakeholders, which has significant limitations. In addition, there are no studies that use evolutionary game model to coordinate the decision-making of various stakeholders and establish a framework for the promotion and application of deep-sea aquaculture under multi-party coordination. (2) The top level design category of existing studies simply emphasizes the important role of government in the development of deep-sea aquaculture, but ignores the cost burden of government in the process and the potential drawbacks of government intervention. (3) Other factors affecting the promotion and application of deep-sea aquaculture have not been analyzed in depth.

Therefore, this study first utilizes an evolutionary game theory model to overcome the limitations of considering a single stakeholder, instead comprehensively analyzing the interactions and strategic evolution among aquaculture enterprise, service organization, and government during the promotion and application of deep-sea aquaculture. Second, while acknowledging the role of government, this study considers the cost issues and government failure issues associated with the promotion and application of deep-sea aquaculture, aiming to identify the threshold for government intervention and achieve an effective combination of government intervention and market mechanisms, thereby realizing the promotion and application of deep-sea aquaculture. Finally, this study fully considers the importance of initial intention, risk, benefit, and fairness in the promotion and application of deep-sea aquaculture to enrich the research conclusions.

## 2 Model construction and analysis

Traditional game theory usually assumes that people are perfectly rational and that participants make decisions under conditions of complete information. However, in real economic life, the conditions of complete rationality and complete information of participants are difficult to achieve. For example, in the competition of enterprises, where there are differences between the participants, the problem of incomplete information and limited rationality due to the economic environment and the complexity of the game problem itself is obvious. Unlike traditional game theory, evolutionary game theory assumes that participants are imperfectly rational and does not require a condition of perfect rationality on the part of the participants. Evolutionary game theory combines game theoretical analysis with dynamic evolutionary process analysis, emphasizing dynamic equilibrium (38).

**Figure 1:**
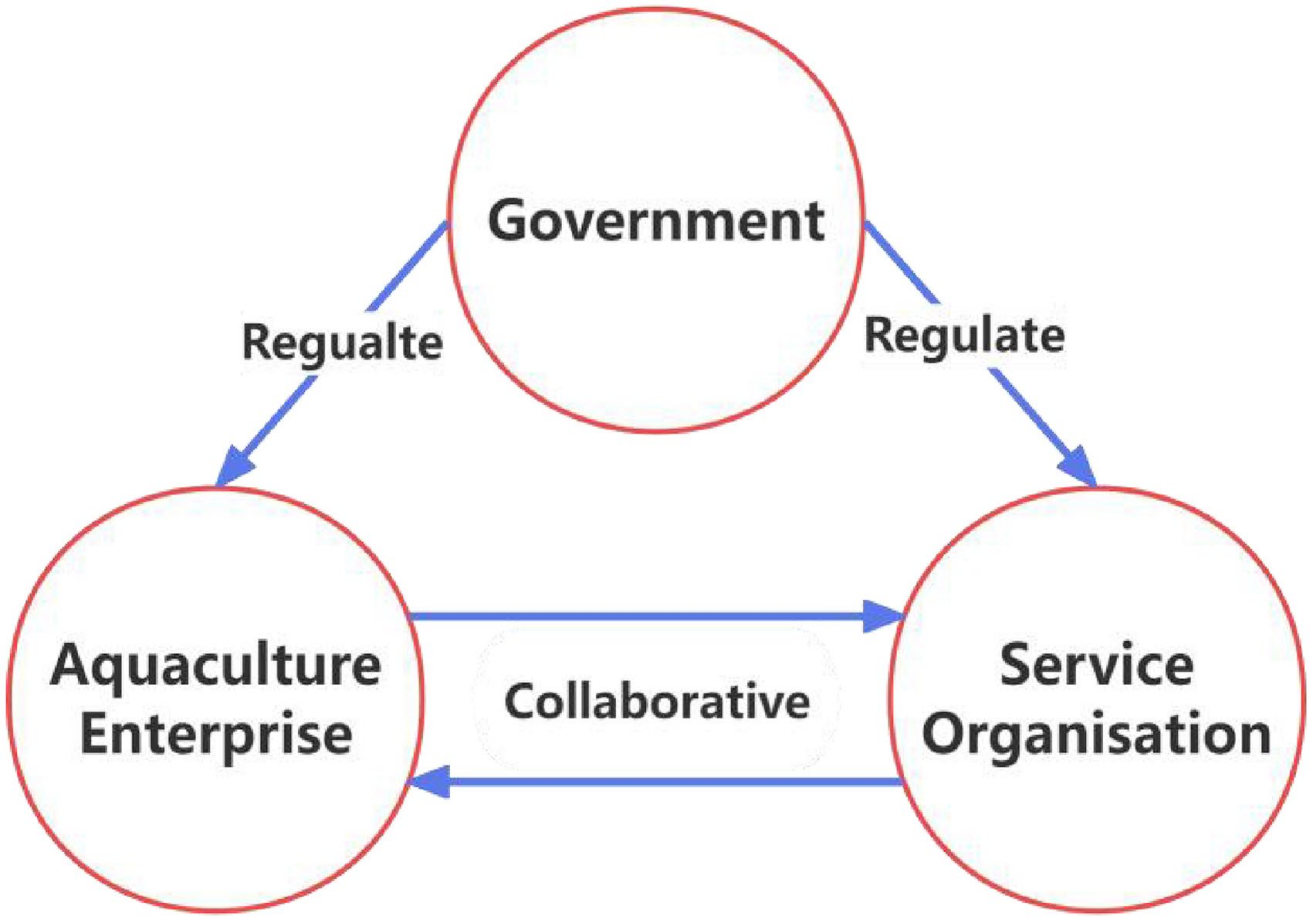
Evolutionary game model diagram.

Deep-sea aquaculture is different from ordinary fishery farming, the input cost is large, so in reality it is very difficult for fishermen to participate in it, and only aquaculture enterprises with a certain scale and economic strength are able to withstand the risk and bear the cost to participate in deep-sea aquaculture. During the aquaculture process, enterprises involved in deep-sea aquaculture interface with relevant service organizations, while the government plays a guiding, supervisory and supportive role (19). It is worth noting that deep-sea aquaculture service providers are mainly relevant scientific research institutes, as well as private or state-owned enterprises, and that the service providers have a profit-making objective in cooperation with enterprises. Deep-sea aquaculture service organizations also face risk when participating in the promotion and application of deep-sea aquaculture. The main risk factors include the high cost of deep-sea aquaculture technology research and development, which may result in insufficient research funding for service organizations. At the same time, the core objectives of service organizations are knowledge innovation and publication of research results, while the core objectives of aquaculture enterprises are cost reduction and profit increase. There are differences in the cooperation objectives of the two parties, and without government leadership and guidance, the connection between the two parties is weak. In general, in the process of promoting and applying deep-sea aquaculture, enterprises bear the responsibility for deep-sea aquaculture; service organizations make cooperation agreements with enterprises and are responsible for technical support; and the government plays a macro-control role (39). Therefore, based on the evolutionary game theory and referencing existing research (40, 41), this study constructs a three-party evolutionary game model of “Aquaculture enterprise—Service organization-Government” under the joint action of market mechanism and government guidance. The relevant model is shown in Figure 1.

### 2.1 Evolutionary game model of “aquaculture enterprise—service organization”

Before constructing the three-party evolutionary game model of “Aquaculture enterprise-Service organization-Governmen,” in order to verify the importance of government involvement, this study firstly constructs a two-party evolutionary game model of “Aquaculture enterprise-Service organization” to investigate the strategy evolution of the two players in the absence of government involvement.

#### 2.1.1 Assumptions of the two-party evolutionary game model of “aquaculture enterprise—service organization”

*H1*: Under the condition of market mechanism, the evolutionary game model for the promotion and application of deep-sea aquaculture should be a two-party evolutionary game model, which is the aquaculture enterprise (Q) and the related service organization (W). The relationship between them is cooperative, with aquaculture enterprise requesting and paying for services related to deep-sea aquaculture from the relevant service organization. In accordance with the principle of maximizing the benefits of each participant, in the model, aquaculture enterprise will choose whether to implement deep-sea aquaculture or not, and relevant service organization will choose whether to provide deep-sea aquaculture service support for aquaculture enterprise or not.

*H2*: Based on Hypothesis 1, the strategy combinations of aquaculture enterprise is whether to engage in deep-sea aquaculture or not, respectively, the strategy space is *α* = (α_1_, α_2_), and this study hypothesizes that the willingness of aquaculture enterprise to participate in deep-sea aquaculture (α_1_) is X, while the willingness not to participate in deep-sea aquaculture (α_2_) is 1-X, and X ∈ [0.1]. The strategy combination of the service organization is whether to provide deep-sea aquaculture services or not, and the strategy space is *β* = (β_1_, β_2_). In this study, it is assumed that the willingness of the service organization to provide deep-sea aquaculture services (β_1_) is Y and the willingness not to provide deep-sea aquaculture services (β_2_) is 1-Y, Y ∈ [0.1].

*H3*: Deep-sea aquaculture enterprise usually have a certain foundation in the aquaculture industry, and service organization also provides services related to offshore aquaculture before providing deep-sea aquaculture services. Therefore, this study assumes that aquaculture enterprise and service organization are not choosing between offshore aquaculture and deep-sea aquaculture, but choosing whether to select deep-sea aquaculture or not on the basis of offshore aquaculture. Based on the cost–benefit function, this study assumes that the cost of implementing offshore aquaculture in aquaculture enterprises is C1 and the benefit of implementing offshore aquaculture is R1; the cost of providing services for offshore aquaculture in service organization is C2 and the benefit of providing services for offshore aquaculture is R2. It is important to note that R1 > C1, R2 > C2. After participating in deep-sea aquaculture, the additional cost of deep-sea aquaculture to be paid by the aquaculture enterprise is C3, the additional cost to be paid by the service organization is C4, the benefit from the implementation of deep-sea aquaculture by the aquaculture enterprise is R3, and the benefit from the service organization’s provision of deep-sea aquaculture services is R4. It is important to note that R3 > C3, R4 > C4. In addition, engaging in deep-sea aquaculture involves a high degree of uncertainty and risk, because of the extreme sea weather that deep-sea aquaculture faces. Therefore, this study assumes that the risk cost of implementing deep-sea aquaculture is C, and that a single individual is likely to be unable to bear the entire risk cost independently. The corresponding parameter relationship can be expressed as: R3-C3-C < 0; R4-C4-C < 0。Because of the cooperative relationship between aquaculture enterprise and service organization, both of them share the risk in the actual process of cooperation. This study assumes that the cost of deep-sea aquaculture risk undertaken by aquaculture enterprise is C7 and the cost of deep-sea aquaculture risk undertaken by service organization is C8, C=C7 + C8. When they cooperate, they can share the cost of risk, in which case R3-C3-C7 > 0; R4-C4-C8 > 0. At the same time, deep-sea aquaculture has the characteristics of high risk and high return, so the average return of the implementation of deep-sea aquaculture should be greater than the average return of offshore aquaculture, in order to facilitate the calculation, this study set the total return of deep-sea aquaculture is higher than the total return of offshore aquaculture, and the specific relationship can be expressed as follows: R3-C3-C7 > R1-C1; R4-C4-C8 > R2-C2. In addition, the risk cost coefficient is T, C7 = T * C, C8 = (1 − T) * C, and T ∈ [0.1].

*H4*: Collaboration between aquaculture enterprise and service organization not only satisfy their respective needs, but also generate benefit from the collaboration. These benefits include resource sharing, cost saving and risk sharing. Therefore, this study assumes that the cooperation benefit from the implementation of deep-sea aquaculture by the aquaculture enterprise and the service organization is R. If one of the parties does not participate in deep-sea aquaculture, there is no cooperation benefit. The coefficient of distribution of the cooperation benefit is A. The cooperation benefit obtained by the aquaculture enterprise is R*A, and the cooperation benefit obtained by the service organization is R*(1-A), A ∈ [0.1].

The detailed parameter list is shown in Table 2.

**Table 2:** Parameters of the two-party evolutionary game model.

Parameter	Parameter name	Exegesis
X	Willingness to participate in deep-sea aquaculture	0 ≤ X ≤ 1
Y	Willingness to provide deep-sea aquaculture services	0 ≤ Y ≤ 1
C1	Cost of offshore aquaculture in aquaculture enterprise	C1 < R1; C1 < C3
C2	Cost of offshore aquaculture service in service organization	C2 < R2; C2 < C4
C3	Cost of deep-sea aquaculture in aquaculture enterprise	C3 < R3; C1 < C3
C4	Cost of deep-sea aquaculture service in service organization	C4 < R4; C2 < C4
C7	Cost of deep-sea aquaculture risk in aquaculture enterprise	C=C7 + C8
C8	Cost of risk in deep-sea aquaculture in service organization	C=C7 + C8
C	Total cost of risk	C=C7 + C8
T	Risk cost coefficient	0 ≤ T ≤ 1
R1	Benefit of offshore aquaculture in aquaculture enterprise	R1 < R3; C1 < R1
R2	Benefit of offshore aquaculture service in service organization	R2 < R4; C2 < R2
R3	Benefit of deep-sea aquaculture in aquaculture enterprise	R1 < R3; C3 < R3
R4	Benefit of deep-sea aquaculture service in service organization	R2 < R4; C4 < R4
R	Benefit of deep-sea aquaculture cooperation	–
A	Cooperation benefit coefficient	0 ≤ A ≤ 1

#### 2.1.2 Analysis of the two-party evolutionary game model of “aquaculture enterprises—service organizations.”

(1) Expected return function and replication of dynamic equation of aquaculture enterprise.

Based on the payment matrix in Table 3, the expected return function for each decision combination of the aquaculture enterprise can be solved, assuming that the expected return function of the aquaculture enterprise participating in deep-sea aquaculture is V_x11_, and that the expected return function of the aquaculture enterprise not participating in deep-sea aquaculture is V_x21_, and that the average expected return is V_x_. Specifically, see Equation (1).


(1) $$\left\{ \begin{array}{l} \begin{array}{l} V_{x11}=(A^{*}R+R1+R3-C1-C3-C7)*y\\ +(R1+R3-C1-C3-C)*(1-y) \end{array}\\ V_{x21}=(R1-C1)*y+(R1-C1)*(1-y)\\ {\bar{V}}_{x}=x^{*}V_{x11}+(1-x)*V_{x21} \end{array}\right.$$

**Table 3:** Payment matrix of the two-party evolutionary game model.

		Aquaculture enterprise	
		Participation	Non-participation
Service organization	Participation	A*R + R1 + R3-C1-C3-C7 (1-A)*R + R2 + R4-C2-C4-C8	R1-C1 R2 + R4-C2-C4-C
	Non-participation	R1 + R3-C1-C3-C R2-C2	R1-C1 R2-C2

Based on the expected return function of the aquaculture enterprise, the dynamic equation of replication of the aquaculture enterprise can be derived, as shown in Equation (2).


(2) $$F(x)=\frac{\mathrm{dx}}{\mathrm{dt}}=x^{*}(x-1)*(C+C3-R3-C^{*}y+C7^{*}y-A^{*}R^{*}y)$$

(2) Expected return function and replication dynamic equation for service organization.

It is assumed that the expected return function for service organizations involved in deep-sea aquaculture is V_y11_, the expected return function for not participating in deep-sea aquaculture is V_y21_, and the average expected return is V_y_. Specifically, see Equation (3).


(3) $$\left\{ \begin{array}{l} \begin{array}{l} V_{y11}=[(1-A)*R+R2+R4-C2-C4-C8]*x\\ +(R2+R4-C2-C4-C)*(1-x) \end{array}\\ V_{y21}=(R2-C2)*x+(R2-C2)*(1-x)\\ {\bar{V}}_{y}=y^{*}V_{y11}+(1-y)*V_{y21} \end{array}\right.$$

Based on the expected return function of the service organization, the replication dynamic equation of the service organization can be derived, as shown in Equation (4).


(4) $$\begin{array}{l} F(y)=\frac{\mathrm{dy}}{\mathrm{dt}}=y^{*}(y-1)*\\ \;\;(C+C4-R4-C^{*}x+C8^{*}x-R^{*}x+A^{*}R^{*}x) \end{array}$$

#### 2.1.3 Stability point analysis of the two-party evolutionary game model of “aquaculture enterprise- service organization”

According to Equation (2) and Equation (4), the Jacobi matrix of the two-party evolutionary game model of “Aquaculture enterprise- Service organization” can be constructed. So that F (x) = 0; F (y) = 0, can find the equilibrium point of the two-party evolutionary game model. The Jacobi matrix of the two-party evolutionary game model of “Aquaculture enterprise- Service organization” is shown in Equation (5).


(5) $$J_{1}=[\begin{array}{l} 0,-x^{*}(x-1)*(C-C7+A^{*}R)\\ -y^{*}(y-1)*(C-C8+R-A^{*}R),0 \end{array}]$$

Based on existing research, according to Lyapunov’s first method (42), equilibria are asymptotically stable if all eigenvalues of the Jacobi matrix have a negative real part, while these equilibria can be called stable points (ESS) only if they satisfy strict Nash equilibrium and pure strategy equilibrium. The equilibrium point is unstable if at least one of the eigenvalues of the Jacobi matrix has a positive real part. The stability of the equilibrium point cannot be determined if the Jacobi matrix has negative real parts except for the eigenvalues that have a real part of zero. Four equilibrium points of the two-party evolutionary game model of “Aquaculture enterprise- Service organization” were calculated: E1 (0,0), E2 (1,0), E3 (0,1), and E4 (1,1). At this time the four equilibrium points constitute the boundary of the evolutionary game domain, the equilibrium points within the boundary are mixed strategy Nash equilibrium, but none of them are stable equilibrium points, so this study focuses on the asymptotic stability of the four boundary equilibrium points mentioned above in the two-party evolutionary game. After substituting each of the above four equilibria into the Jacobi matrix, the eigenvalues are derived, as shown in Table 4.

**Table 4:** Eigenvalues of Jacobi matrix.

ESS	λ1	λ2
E1(0,0)	R3 - C3 - C	R4 - C4 - C
E2(1,0)	C + C3 - R3	R - C8 - C4 + R4 - A*R
E3(0,1)	C + C4 - R4	R3 - C7 - C3 + A*R
E4(1,1)	C3 + C7 - R3 - A*R	C4 + C8 - R4 -(1-A)*R

Based on the assumptions of this study, it can be seen that there are two stable points in the two-party evolutionary game model of “Aquaculture enterprise-Service organization,” which are E1 (0, 0) and E4 (1, 1). It can be seen that, without government participation and relying only on the market mechanism, the model is unable to reach the equilibrium point E4 in a stable manner, which is due to the fact that the cost of deep-sea aquaculture is too high, and the aquaculture enterprise and service organization are unwilling to take the risk, and then relying only on the market mechanism will lead to the phenomenon of market failure. Therefore, in order to study the importance of government participation, this study continues to construct a three-party evolutionary game model of “Aquaculture enterprise- Service organization-Government” to investigate the stability of the model.

### 2.2 A three-party evolutionary game model of “aquaculture enterprise- service organization-government”

#### 2.2.1 Assumptions of the three-party evolutionary game model of “aquaculture enterprise- service organization-government”

*H5*: Because of the high cost of inputs required for deep-sea aquaculture, it is difficult to rely only on enterprise and service organization, and in order to reduce the risk of each participant and increase the willingness to participate, the government (E) needs to play a macro-control role and provide support for aquaculture enterprise and related service organization. Therefore, the government’s strategic combination is whether to provide deep-sea aquaculture support or not. In summary, this study constructed a three-party evolutionary game model of “Aquaculture enterprise- Service organization-Government” on the basis of the two-party evolutionary game model of “Aquaculture enterprise-Service organization.” The government’s strategy portfolio is whether to provide deep-sea aquaculture support or not, and the strategy space is *θ* = (θ_1_, θ_2_). In this study, it is assumed that the government’s willingness to provide deep-sea aquaculture support (θ_1_) is Z, and its willingness not to provide deep-sea aquaculture support (θ_2_) is 1-Z, Z ∈ [0.1].

*H6*: In order to increase the motivation of aquaculture enterprise and service organization to participate in deep-sea aquaculture, the government will give subsidies to aquaculture enterprise and service organization at the beginning. Assume that the government grants deep-sea aquaculture subsidies to the aquaculture enterprise is S1, and grants deep-sea aquaculture subsidies to the service organization is S2, the total subsidy is S, and the subsidy coefficient is T. The specific relationship can be expressed as follows: S1 = S*T, S2 = S*(1-T), and T∈[0.1]. In addition, the government likewise plays a supervisory role by imposing penalties if a party is found to be in breach of contract, i.e., when one party adopts a positive cooperation strategy while the other adopts a negative cooperation strategy, the penalty is K1 for the enterprise and K2 for the service organization.

Scenario 7: Both general and deep-sea aquaculture place a burden on the environment. While environmental regulations, such as discharge permits, internalize private costs so that enterprises bear the costs of environmental damage, the government also bears the environmental costs of environmental damage caused by economic development. It is important to note that this study incorporates the privately borne compensation for environmental damage into the cost of business. In summary, this study assumes that the government will bear the environmental cost of offshore aquaculture as C5, and the environmental cost of deep-sea aquaculture as C6. In addition, deep-sea aquaculture will to a certain extent play a role in ecological restoration, and therefore it is assumed that the environmental benefits of deep-sea aquaculture will be R5, which belongs to the positive externality benefits that cannot be appropriated by the aquaculture enterprise, and the government ultimately appropriates them. Meanwhile, in the process of providing deep-sea aquaculture service, service organization will generate relevant patents or intellectual property rights (such as research and development of seedling and development of new types of equipment), and although these benefits are incorporated into the service organization, they will potentially generate benefits related to the national strategic level and improve the country’s international status, so this study assumes that the strategic benefits brought by the provision of deep-sea aquaculture service is R6.

The detailed parameter list and payment matrix are shown in Tables 5, 6.

**Table 5:** Parameters of the three-party evolutionary game model.

Parameter	Parameter name	Exegesis
X	Willingness to participate in deep-sea aquaculture	0 ≤ X ≤ 1
Y	Willingness to provide deep-sea aquaculture services	0 ≤ Y ≤ 1
Z	Willingness to support deep-sea aquaculture	0 ≤ Z ≤ 1
C1	Cost of offshore aquaculture in aquaculture enterprise	C1 < R1; C1 < C3
C2	Cost of offshore aquaculture service in service organization	C2 < R2; C2 < C4
C3	Cost of deep-sea aquaculture in aquaculture enterprise	C3 < R3; C1 < C3
C4	Cost of deep-sea aquaculture service in service organization	C4 < R4; C2 < C4
C5	Environmental cost of offshore aquaculture	C5 > C6
C6	Environmental cost of deep-sea aquaculture	C5 > C6; C6 < R5
C7	Cost of deep-sea aquaculture risk in aquaculture enterprise	C=C7 + C8
C8	Cost of risk in deep-sea aquaculture in service organization	C=C7 + C8
C	Total cost of risk	C=C7 + C8
T	Risk cost coefficient	0 ≤ T ≤ 1
R1	Benefit of offshore aquaculture in aquaculture enterprise	R1 < R3; C1 < R1
R2	Benefit of offshore aquaculture service in service organization	R2 < R4; C2 < R2
R3	Benefit of deep-sea aquaculture in aquaculture enterprise	R1 < R3; C3 < R3
R4	Benefit of deep-sea aquaculture service in service organization	R2 < R4; C4 < R4
R5	Environmental benefit of deep-sea aquaculture	C6 < R5
R6	Strategic benefit of deep-sea aquaculture	–
R	Benefit of deep-sea aquaculture cooperation	–
A	Cooperation benefit coefficient	0 ≤ A ≤ 1
S1	Subsidies for deep-sea aquaculture in aquaculture enterprise	S=S1 + S2
S2	Subsidies for deep-sea aquaculture in service organization	S=S1 + S2
K1	Penalties for aquaculture enterprise	–
K2	Penalties for service organization	–
S	Deep-sea aquaculture total subsidies	S=S1 + S2
B	Subsidy coefficient	0 ≤ B ≤ 1

**Table 6:** Payment matrix for the three-party evolutionary game model.

		Government	
		Support: Z	Not support:1-Z
Aquaculture enterprise involved in deep-sea aquaculture: X	Service organization involved in deep-sea aquaculture: Y	A*R + R1 + R3-C1-C3 + B*S-C7 (1-A)*R + R2 + R4-C2-C4 + (1-B)*S-C8 R5 + R6-C5-C6-S	A*R + R1 + R3-C1-C3-C7 A*R + R2 + R4-C2-C4-C8 R5 + R6-C5-C6
	Service organization not involved in deep-sea aquaculture:1-Y	R1 + R3-C1-C3-C + S1	R1 + R3-C1-C3-C
		R2-C2-K2	R2-C2
		R5-C5-C6-S1-S3	R5-C5-C6
Aquaculture enterprise not involved in deep-sea aquaculture:1-X	Service organization involved in deep-sea aquaculture: Y	R1-C1-K1	R1-C1
		R2 + R4-C2-C4-C + S2	R2 + R4-C2-C4-C
		R6-C5-S2 + K1	R6-C5
	Service organization not involved in deep-sea aquaculture:1-Y	R1-C1-K1	R1-C1
		R2-C2-K2	R2-C2
		-C5 + K1 + K2	-C5

#### 2.2.2 Analysis of the three-party evolutionary game model of “aquaculture enterprise- service organization-government.”

(1) Expected return function and replication dynamic equation of aquaculture enterprise.

Based on the payment matrix in Table 6, the expected return function for each decision combination of the aquaculture enterprise can be solved, assuming that the expected return function of the aquaculture enterprise participating in deep-sea aquaculture is V_x11_, and that the expected return function of the aquaculture enterprise not participating in deep-sea aquaculture is V_x21_, with an average expected return of V_x_. Specifically, it is shown in Equation (6).


(6) $$\left\{ \begin{array}{l} V_{\chi 11}={\left(A^{*}R+R1+R3-C1-C3+B^{*}S-C7\right)}^{*}y^{*}z+\\ {\left(A^{*}R+R1+R3-C1-C3-C7\right)}^{*}y^{*}(1-z)+\\ {\left(R1+R3-C1-C3-C+S1+S3\right)}^{*}{\left(1-y\right)}^{*}z+\\ {\left(R1+R3-C1-C3-C\right)}^{*}{\left(1-Y\right)}^{*}(1-z)\\ V_{\chi 21}={\left(R1-C1\right)}^{*}y^{*}z+{\left(R1-C1\right)}^{*}y^{*}(1-z)+\\ {\left(R1-C1\right)}^{*}{\left(1-y\right)}^{*}z+{\left(R1-C1\right)}^{*}{\left(1-y\right)}^{*}(1-z)\\ {\bar{V}}_{\chi }=\chi^{*}V_{\chi 11}+(1-\chi )*V_{\chi 21} \end{array}\right.$$

Based on the expected return function of the aquaculture enterprise, the replication dynamic equation of the aquaculture enterprise can be obtained as, as shown in Equation (7).


(7) $$\begin{array}{l} F(x)=\frac{\mathrm{dx}}{\mathrm{dt}}=-x^{*}(x-1)*(R3-C3-C+C^{*}y-C7^{*}y+\\ \;K1^{*}z+S1^{*}z+A^{*}R^{*}y-S1^{*}y^{*}z+S^{*}B^{*}y^{*}z) \end{array}$$

(2) Expected return function and replication dynamic equation of service organizations.

It is assumed that the expected return function of service organization participating in deep-sea aquaculture is V_y11_, the expected return function of not participating in deep-sea aquaculture is V_y21_, and the average expected return is V_y_. Specifically, it is shown in Equation (8).


(8) {Vy11=[(1−A)∗R+R2+R4−C2−C4+(1−B)∗S−C8)∗x∗z+[(1−A)∗R+R2+R4−C2−C4−C8)A∗x∗(1−z)+(R2+R4−C2−C4−C+S2)∗(1−x)∗z+(R2+R4−C2−C4−C)∗(1−x)∗(1−z)Vy21=(R2−C2−K2)∗x∗z+(R2−C2)∗x∗(1−z)+(R2−C2−K2)∗(1−x)∗z+(R2−C2)∗(1−x)∗(1−z)V¯y=y∗Vy11+(1−y)∗Vy21

Based on the expected return function of the service organization, the dynamic equation of replication of the service organization can be derived, as shown in Equation (9).


(9) $$\begin{array}{l} F(y)=\frac{\mathrm{dy}}{\mathrm{dt}}=y^{*}(y-1)*(C+C4-R4-C^{*}x+C8^{*}x-K2^{*}z-\\ R^{*}x-S2^{*}z+A^{*}R^{*}x-S^{*}x^{*}z+S2^{*}x^{*}z+S^{*}B^{*}x^{*}z) \end{array}$$

(3) Expected return function and replication dynamic equation for the government.

Assuming that the expected return function for government support for deep-sea aquaculture is Vz11, the expected return function for no support for deep-sea aquaculture is Vz21, and the average expected return is Vz. Specifically it is shown in Equation (10).


(10) {VZ11=(R5+R6−C5−C6−S)∗x∗y+R5−C5−C6−S1+K2)A∗x∗(1−y)+(R6−C5−S2+K1)∗(1−x)∗y+(−C5+K1+K2)∗(1−x)∗(1−y)VZ21=(R5+R6−C5−C6)∗x∗y+(R5−C5−C6)∗x∗(1−y)+(R6−C5)∗(1−x)∗y−C5∗(1−x)∗(1−y)V¯Z=z∗VZ11+(1−z)∗VZ21

Based on the government’s expected return function, the government’s dynamic equation for replication can be derived, as shown in Equation (11).


(11) $$\begin{array}{l} F(z)=\frac{\mathrm{dz}}{\mathrm{dt}}=z^{*}(z-1)*(K1^{*}x-K2-K1+K2^{*}y+\\ S1^{*}x+S2^{*}y+S^{*}x^{*}y-S1^{*}x^{*}y-S2^{*}x^{*}y) \end{array}$$

#### 2.2.3 Analysis of the stability point of the three-party evolutionary game model of “aquaculture enterprise- service organization- government”

According to Equation (7), Equation (9), Equation (11), this study can construct the Jacobi matrix of the three-party evolutionary game model of “Aquaculture enterprise- Service organization-Government,” make F (x) = 0; F (y) = 0, F (z) = 0 can be obtained the equilibrium point of the three-party evolutionary game model. Due to the three-party evolutionary game Jacobi matrix is too long, so the study uses symbols instead, Jacobi matrix as shown in Equation (12).


(12) $$\left[\begin{array}{l} \text{diff}(\mathrm{fx},x)\;\;\text{diff}(\mathrm{fx},y)\;\;\text{diff}(\mathrm{fx},z)\\ \text{diff}(\mathrm{fy},x)\;\;\text{diff}(\mathrm{fy},y)\;\;\text{diff}(\mathrm{fy},z)\\ \text{diff}(\mathrm{fz},x)\;\;\text{diff}(\mathrm{fz},y)\;\;\text{diff}(\mathrm{fz},z) \end{array}\right]$$

The three-party evolutionary game model of “Aquaculture enterprise- Service organization-Government” has eight pure strategy equilibrium points: E1(0,0,0), E2(1,0,0), E3(0,1,0), E4(0,0,1), E5(1,1,0), E6(1,0,1), E7(0,1,1), E8(1,1,1). Substituting the equilibrium points into the Jacobi matrix, respectively, leads to the eigenvalues as shown in Table 7. According to the assumptions of this study, the sign of the eigenvalues of the equilibrium points of the Jacobi matrix is shown in Table 8.

**Table 7:** Eigenvalues of the Jacobi matrix for the three-party evolutionary game model.

ESS	λ1	λ2	λ3
E1(0,0,0)	R3 - C3 - C	R4 - C4 - C	K1 + K2
E2(1,0,0)	C + C3 - R3	R - C8 - C4 + R4 - A*R	K2 - S1
E3(0,1,0)	R3 - C7 - C3 + A*R	C + C4 - R4	K1 - S2
E4(0,0,1)	K1 - C3 - C + R3 + S1	K2 - C4 - C + R4 + S2	- K1 - K2
E5(1,1,0)	C3 + C7 - R3 - A*R	C4 + C8 - R - R4 + A*R	-S
E6(1,0,1)	C + C3 - K1 - R3 - S1	K2 - C8 - C4 + R + R4 + S - S*B - A*R	S1 - K2
E7(0,1,1)	K1 - C7 - C3 + R3 + S*B + A*R	C + C4 - K2 - R4 - S2	S2 - K1
E8(1,1,1)	C3 + C7 - K1 - R3 - S*B - A*R	C4 + C8 - K2 - R - R4 - S + S*B + A*R	S

**Table 8:** Signs of the eigenvalues of the equilibrium points of the three party evolutionary game model.

Equilibrium point	Eigenvalues translation			
	λ1	λ2	λ3	Stability
E1(0,0,0)	−	−	+	NESS
E2(1,0,0)	+	+	×	NESS
E3(0,1,0)	+	+	×	NESS
E4(0,0,1)	×	×	−	NESS
E5(1,1,0)	−	−	−	ESS
E6(1,0,1)	−	+	×	NESS
E7(0,1,1)	+	×	×	NESS
E8(1,1,1)	×	×	+	NESS

× stands for sign uncertainty, ESS stands for stable point and NESS stands for non-stable point.

According to Table 8, it can be seen that based on the assumptions of this study, compared with the two-party evolutionary game model, the equilibrium point of the three-party evolutionary game model is E5 (1, 1, 0), and is not affected by the value of the parameter, and it is always a stable point. E1 (0, 0, 0) cannot be a stable point, which indicates that the government’s intervention and support effectively guarantees the promotion and application of deep-sea aquaculture.

In addition, the following clarifications are needed in this study:

(1) According to the eigenvalues of Jacobi matrix, it can be seen that in order to avoid the failure of government intervention, which leads to E4(0,0,1) becoming the equilibrium point, the government’s participation in the process of promoting the application of deep-sea aquaculture has three measure options. First, good subsidy measures, the subsidy should be greater than or equal to the risk cost borne by a single individual independently engaging in deep-sea aquaculture, the specific relationship can be expressed as: S1 ≥ R3-C3-C or S2 ≥ R4-C4-C. Second, strengthen the supervision, and strengthen the punishment of violators of cooperation or those who are negatively treated, and the amount of punishment should be greater than or equal to the risk cost borne by a single individual independently engaging in deep-sea aquaculture, and the specific relationship can be expressed as K1 ≥ R3-C3-C or K2 ≥ R4-C4-C. Third, the government can also act on subsidies and penalties at the same time, so that the sum of the penalty amount and the subsidy amount is greater than or equal to the risk cost borne by a single individual independently engaging in deep-sea aquaculture.
(2) The three-party evolutionary game model can never be stabilized at E8 (1, 1, 1), which indicates that the government cannot continuously intervene in the process of deep-sea aquaculture promotion and application. The main reasons are: firstly, the special characteristics of deep-sea aquaculture lead to high input costs, high technological uncertainty, and large subsidies required, and sustained subsidies will exacerbate the government’s financial burdens and increase the government’s fiscal deficits. Secondly, in the short term, government intervention, especially policy subsidies, will reduce the initial investment risk of deep-sea aquaculture and stimulate the rapid expansion of the deep-sea aquaculture industry. However, in the long term, as the scale of the industry expands, the marginal benefit of government subsidies will decrease, but the government’s financial burden will continue to rise, and even the enterprise will rely on government subsidies to maintain its operation rather than to improve the efficiency of its operation, thus creating the phenomenon of “policy parasitism.” Finally, the continued imposition of penalties by the government, although it will force aquaculture enterprise and service organization to participate in deep-sea aquaculture, is not in line with the rule of the market, and will ultimately lead to a failure of the market and government intervention at the same time. In addition, three-party evolutionary game model can stabilize at E5 (1, 1, 0) indicating that as the deep-sea aquaculture industry continues to develop, aquaculture enterprises and service institutions no longer rely on government subsidies but instead rely on market mechanisms to operate independently, and the need for government intervention is gradually decreasing.

## 3 Simulation analysis

### 3.1 Parameter assignment

In order to more intuitively study the strategy evolution of each participant and more deeply explore the influencing factors in the process of deep-sea aquaculture promotion and application, this study continues to simulate and analysis the evolutionary game model. Based on the previous assumptions, this study assigns values to each parameter, and the detailed parameter assignment table is shown in Table 9.

**Table 9:** Parameter assigned values for the evolutionary game model.

Parameter	C1	C2	C3	C4	C5	C6	C7	C8	C	T
	15	5	20	20	15	10	25	25	50	0.5
	R1	R2	R3	R4	R5	R6	R	A	S1	S2
	20	10	60	60	30	40	30	0.5	10	10
	K1	K2	S	B	x	y	z			
	5	5	20	0.5	0.5	0.5	0.5			

### 3.2 Evolutionary combinatorial diagram analysis

**Figure 2:**
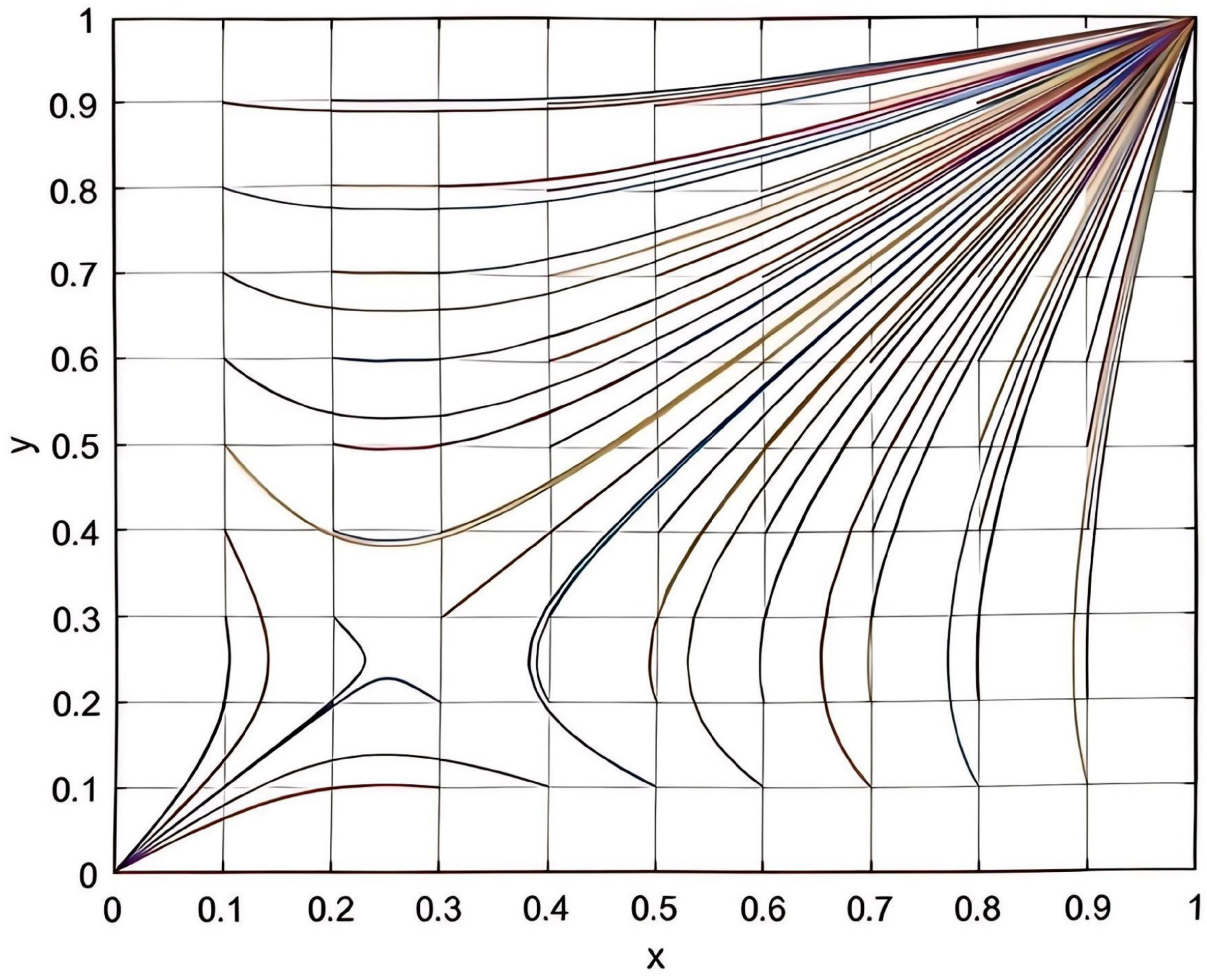
Combination diagram of two-party evolutionary game.

**Figure 3:**
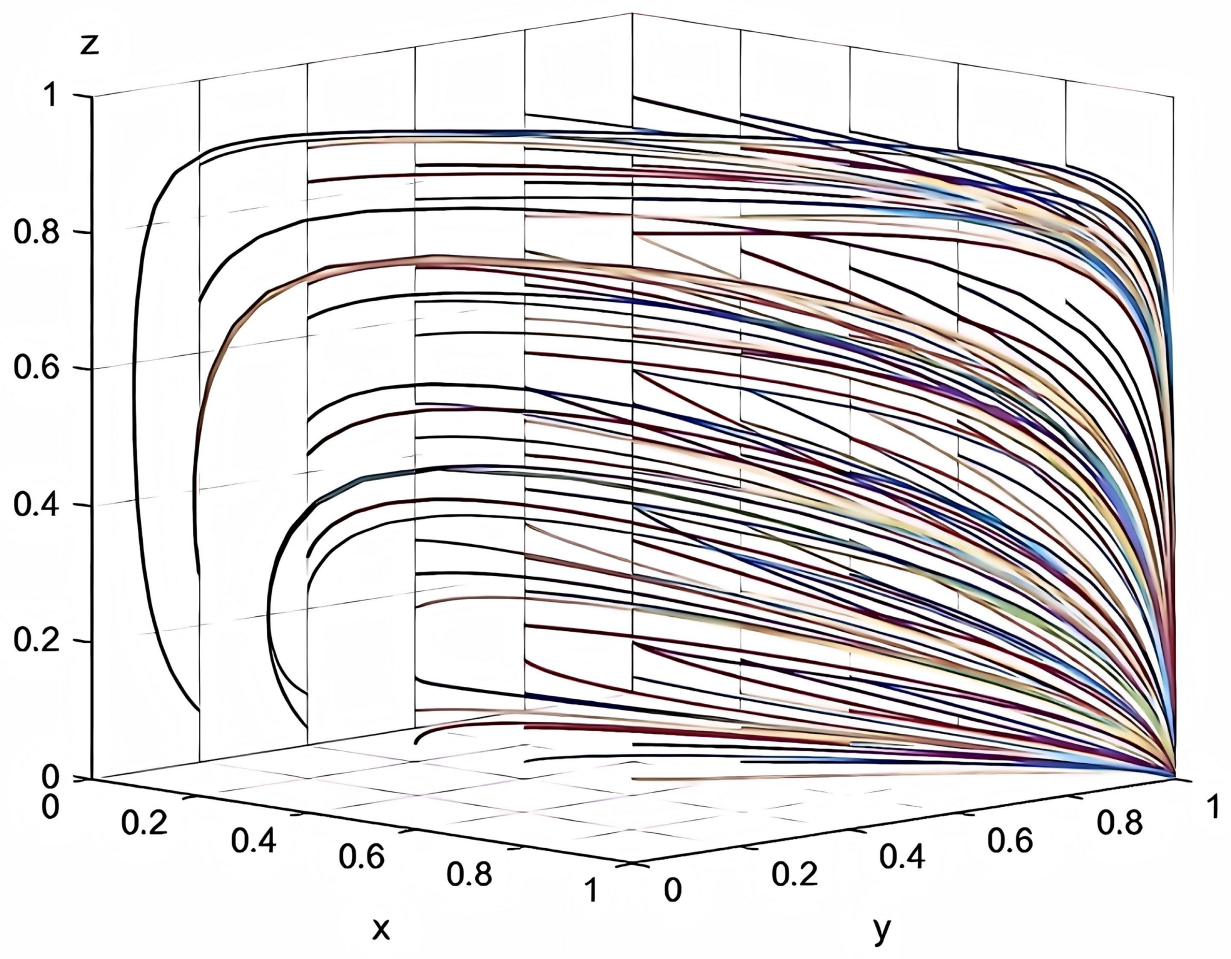
Combination diagram of three-party evolutionary game.

According to the parameters set in this study, this study plots the evolutionary game combinations based on the two-party evolutionary game model and the three-party evolutionary game model established above. See the detail in Figures 2, 3.

According to Figure 2, it can be seen that in the absence of government intervention, there is a tendency of “participation, participation” and “non-participation, non-participation” between enterprise and service organization. Meanwhile, according to Table 3, it can be inferred that as the total cost of risk rises, the participating entities will tend to be more and more inclined to “non-participation, non-participation.” The results of the analysis in Figure 2 are corroborated by the results of the analysis in Table 3. According to Figure 3, it can be seen that the three-party evolutionary game model converges to the stable point E5(1,1,0) after the government intervention is adopted, indicating that the government intervention can effectively improve the willingness to participate in deep-sea aquaculture of aquaculture enterprise and service organization. Meanwhile, according to Figure 3, it can also be seen that in the evolution process, no matter how high the government’s willingness to participate is, it finally converges to the stable point E5, which is corroborated with the results of the analysis in Table 7.

### 3.3 Numerical simulation analysis

#### 3.3.1 Analysis of the impact of initial willingness to participate

**Figure 4:**
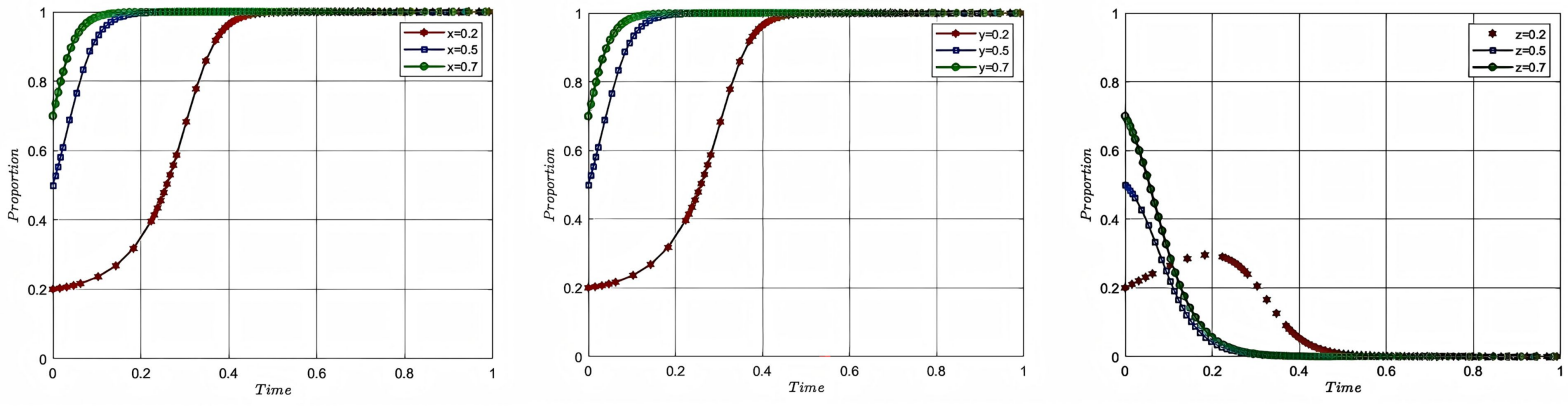
Analysis of the impact of initial willingness.

In order to study the impact of the initial willingness of each participant on the process and outcome of the evolutionary game, this study assigns the initial willingness of aquaculture enterprise, service organization, and government to 0.2, 0.5, and 0.7, with the corresponding colors of red, blue, and green, respectively, while the rest of the parameters remain unchanged, and plots Figure 4. In Figure 4, from left to right, the impact of the change in the initial willingness of each of the aquaculture enterprise, service organization and government on the process and outcome of the evolutionary game is shown respectively, with the x-axis being the evolutionary cycle and the y-axis being the initial willingness to participate.

Figure 4 shows that the stronger the initial willingness of aquaculture enterprise and service organization, the faster they will choose to participate in deep-sea aquaculture. The government, as the initial willingness increases, will tend to 0 at a slower rate, and when the initial willingness of all parties to participate is 0.2, the government’s willingness to participate shows a tendency to rise first and then decrease. The analysis of the impact of initial willingness to participate explains the mechanism of the stage-by-stage evolution of deep-sea aquaculture promotion and application. In the early stage of industrial development, facing the dilemma of negative willingness to participate of both aquaculture enterprise and service organization, the government needs to stimulate the market vitality effectively through institutional intervention. Specifically, through the implementation of intervention strategies, such as financial subsidies and tax incentives to resolve the cost concerns of market entities, and targeted industrial policy push to solve the information asymmetry problem. Along with the initial formation of the cooperation network between aquaculture enterprise and service organization and the gradual improvement of the market mechanism, the intensity of government intervention will be adjusted according to the degree of improvement of the market mechanism, and the role of the government will be changed from a dominant character to a service-oriented character, so as to ultimately realize the smooth transition of the deep-sea aquaculture promotion and application from an administrative-driven approach to a market-driven approach.

#### 3.3.2 Analysis of the impact of deep-sea aquaculture risk

**Figure 5:**
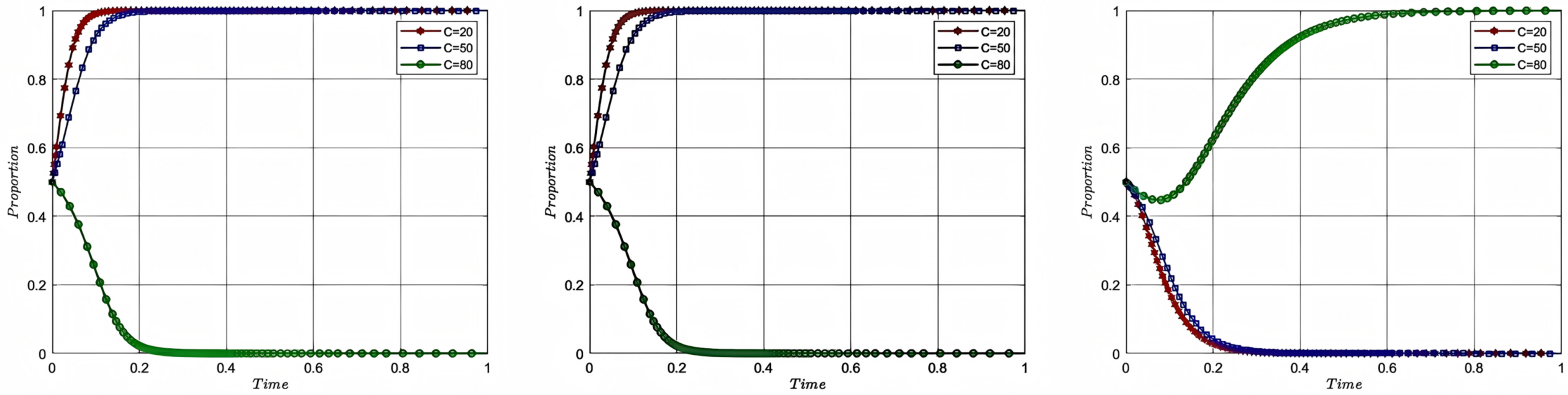
Analysis of the impact of the cost of risk.

**Figure 6:**
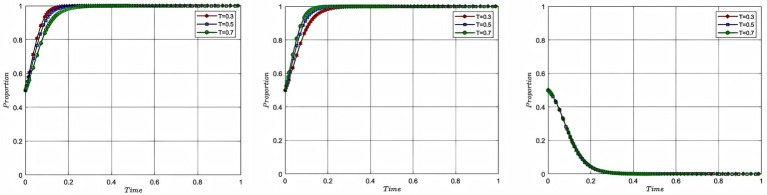
Analysis of the impact of the cost of risk coefficient.

In order to study the impact of risk cost on the process and outcome of the evolutionary game of each participating subject, this study assigns the total risk cost as 20, 50 and 80, with the corresponding colors of red, blue and green, while keeping the rest of the parameters unchanged, and plots Figure 5. In Figure 5, the evolutionary game process and result of the aquaculture enterprise and service organization and the government are shown from left to right, with the x-axis being the evolutionary cycle and the y-axis being the willingness of the participating entities to participate. In order to explore the risk cost coefficient on the evolutionary game process and result of each participating subject, this study assigns the risk cost coefficient as 0.3, 0.5, and 0.7, with corresponding colors of red, blue and green, while keeping the rest of the parameters unchanged, and plots Figure 6. In Figure 6, the evolutionary game process and result of the aquaculture enterprise and service organization and the government are shown from left to right, with the x-axis being the evolutionary cycle and the y-axis being the willingness of the participating entities to participate.

According to Figure 5, it can be seen that as the cost of risk rises, the willingness to participate of each participant changes. When the total risk cost rises from 20 to 50, the speed of convergence of the willingness to participate of the enterprise, service organization and government decreases. When the risk cost becomes 80, the excessive risk cost makes the probability of participants engaging in deep-sea aquaculture suffering from losses too large, aquaculture enterprise and service organization cannot form the motivation to participate, and at this time, the government cannot effectively mobilize the enthusiasm of the both of them through intervention. Therefore, the model evolution result shows that the willingness of aquaculture enterprise and service organization to participate converges to 0, and the willingness of the government to participate converges to 1. The government intervention does not achieve the desired effect, and government failure occurs at this time. According to Figure 6, it can be seen that as the cost of risk taking by aquaculture enterprise rises, the willingness of enterprise to participate tends to 1 slower, while the willingness of service organization to participate tends to 1 faster, and the degree of fit between them decreases. At the same time, there is no impact on the government’s willingness to participate, because the total cost has not changed. The analysis of risk cost of deep-sea aquaculture reveals the key rule of risk transmission in the process of deep-sea aquaculture promotion and application: when the risk cost breaks through the threshold, aquaculture enterprise and service organizations will produce negative psychology due to the “loss aversion effect,” and then the government’s unidirectional intervention triggers the trap of adverse selection, resulting in the failure of the government. When the proportion of risk structure is imbalanced, aquaculture enterprise bears too much risk, the risk transfer mechanism prompts service organization to participate in the enthusiasm, but there is also the phenomenon that aquaculture enterprise and service organization cannot be matched effectively, resulting in the loss of cooperation efficiency.

#### 3.3.3 Analysis of the impact of benefits from deep-sea aquaculture cooperation

**Figure 7:**
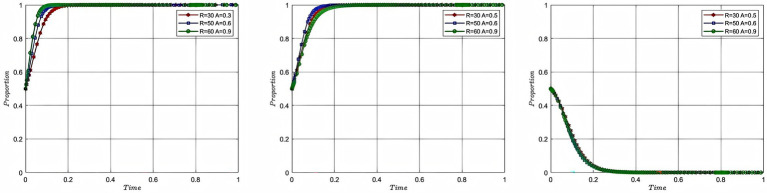
Analysis of the impact of cooperation benefits and distribution coefficient.

In order to study the influence of cooperation benefits on the process and result of the evolutionary game of each participating subject, this study assigns the values of 30, 50, 60 to the total cooperation benefits and 0.5, 0.6, 0.9 to the coefficient of distribution of the cooperation benefits, while keeping the rest of the parameters unchanged, with the corresponding colors of red, blue and green, respectively, and plots Figure 7. In Figure 7, the impacts of the evolutionary gaming process and result of aquaculture enterprise, service organization and government are shown from left to right, with the x-axis showing the evolutionary cycle and the y-axis showing the willingness to participate of the participating entities.

According to Figure 7, it can be seen that the willingness of enterprise to participate tends to 1 at a faster rate as the benefits of cooperation and the coefficient of distribution of the benefits of cooperation among aquaculture enterprise increase. On the contrary, when the cooperation benefits are 30 and the allocation coefficient of the service organization is 0.5, the service organization can get cooperation benefits of 15, and when the cooperation benefits are 50 and the allocation coefficient of the service organization is 0.4, the service organization can get cooperation benefits of 20, so the willingness of the service organization to participate tends to 1 faster. When the cooperation benefits continue to rise to become 60 and the service organization allocation coefficient falls to 0.1, the cooperation benefits available to the service organization at this point are 6, so the willingness to participate tends to 1 at a much lower rate. The government, on the other hand, tends toward a slightly faster willingness to participate of 0 as total cooperation benefits rise, this is because as the benefits of enterprise and service organization rise, motivation increases and the government is able to end its intervention earlier. However, if the distribution of the benefits of cooperation is never equitable, there will also be a mismatch between the willingness of aquaculture enterprise and service organization to participate, and the efficiency of cooperation will be impaired. The analysis of the impacts of cooperation benefits and distribution coefficient reveals the dual driving mechanism of cooperation benefit distribution in the process of deep-sea aquaculture promotion and application. Firstly, the efficiency mechanism: the strategy selection of the participating subjects follows the principle of profit maximization, and the willingness to participate shows a significant positive correlation with the absolute benefits. Secondly, the equity mechanism: when the total cooperation benefit is in conflict with its own allocation coefficient, there is an imbalance in equity, and the reverse driving mechanism is triggered after reaching a critical value, resulting in an impairment of the efficiency of cooperation between the two parties. This essentially reflects the “efficiency-equity” interaction in cooperation, and the driving mechanism needs to satisfy the dual conditions of expansion of the scale of benefits and a reasonable structure of benefit distribution.

#### 3.3.4 Analysis of the combined impact of cost and government intervention

**Figure 8:**
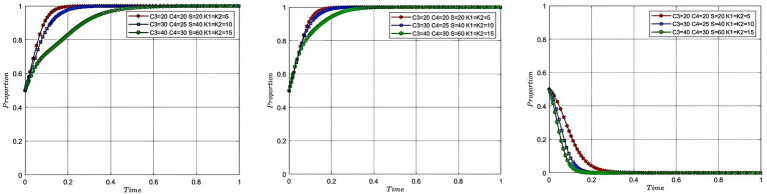
Analysis of the combined impact of cost and government intervention.

In order to investigate the evolution process and result of the strategies of each participant under the combined influence of cost and government intervention. In this study, the cost of deep-sea aquaculture for aquaculture enterprise was assigned as 20, 30, and 40, and the cost of deep-sea aquaculture for service organization was assigned as 20, 25, and 30, respectively, while the rest of the parameters were kept unchanged. The total government subsidy is set to 20, 40 and 60 and the penalties are set to 5, 10, and 15. The corresponding colors are red, blue and green and are plotted in Figure 8. In Figure 8, the impacts of the evolutionary gaming process and result of aquaculture enterprise, service organization and government are shown from left to right, with the x-axis showing the evolutionary cycle and the y-axis showing the willingness to participate of the participating entities.

According to Figure 8, it can be seen that even though the intensity of government intervention and the cost of deep-sea aquaculture rise together, the rate of convergence of aquaculture enterprise and service organization still slows down as the cost rises. And when the cost reaches 60, the speed of convergence decreases substantially. This is because at this point the benefits of participating in deep-sea aquaculture are already negative, and the reason why aquaculture enterprise and service organization continue to converge at 1 is attributed to government intervention. Governments, on the other hand, converge progressively faster as the cost of intervention rises. In summary, this simultaneous rise in cost and subsidy leads to an eventual dilemma where the government bears high costs while failing to increase the willingness of participating entities to participate. The analysis of the combined impact of cost and government intervention shows that every policy tool must comply with the rule of the market, and government intervention that violates the rule of the market will not only lead to the dilemma of double failure of the government and the market, but will also lead to the transformation of aquaculture enterprise and service organization from value creation to subsidy dependence, and the basis of decision-making from profit-driven to policy arbitrage-driven. Analysis of the combined impact of cost and government intervention indicates that any policy tool must conform to market principles. Government intervention that violates market principles not only leads to a situation of double failure of government and market, but also causes aquaculture enterprise and service organization to shift from value creation to subsidy dependence. At this point, the double failure of government and market is mainly manifested in the inability of aquaculture enterprises and service organizations to cooperate through market mechanisms, and the government paying high intervention costs without achieving the expected results.

## 4 Conclusion

The study analyzed the influencing factors in the promotion and application of deep sea aquaculture by constructing a two-party evolutionary game model of “Aquaculture enterprise -Service organization” and a three-party evolutionary game model of “Aquaculture enterprise- Service organization-Government.” The main conclusions are as follow:

(1) By constructing a two-party evolutionary game model of “aquaculture enterprise-service organization” and conducting stability analysis, it is found that the two-party evolutionary game model has two equilibrium points: (0, 0) and (1, 1), and neither participant can stably choose an active participation strategy. After introducing government intervention, the three-party evolutionary game model shows that the three-party evolutionary game model of “aquaculture enterprise–service organization–government” stably converges to (1, 1, 0).
(2) Analysis of the impact of initial willingness to participate shows that the initial willingness of each participating entity to participate significantly affects the strategic choices of aquaculture enterprise and service organization. The stronger the initial willingness, the greater the likelihood that participating entities will actively engage in deep-sea aquaculture. However, when the willingness of aquaculture enterprise and service organization to participate is too low, the willingness of the government to participate shows a trend of first increasing and then decreasing.
(3) Simulation analysis of risk cost and risk coefficient shows that if the risk cost of deep-sea aquaculture is too high, it will cause the willingness of aquaculture enterprise and service organization to participate to tend toward 0. This threshold is when the sum of risk and cost exceeds the sum of potential profits from deep-sea aquaculture and government subsidies received. At this point, the government’s willingness to participate tends toward 1, but it is still unable to effectively promote the effective participation of aquaculture enterprise and service organization in deep-sea aquaculture.
(4) Simulation analysis of cooperation benefit and distribution coefficient shows that an increase in cooperation benefit between aquaculture enterprise and service organization will enhance the willingness of both parties to participate in deep-sea aquaculture through efficiency mechanisms, enabling the government to end intervention more quickly. However, when cooperation benefit distribution is imbalanced, it will lead to a mismatch in the willingness of aquaculture enterprise or service organization to participate.
(5) Simulation analysis under the combined impact of cost and government intervention shows that as the cost of participating in deep-sea aquaculture and government subsidy both rise, and the subsidy increases at a faster rate, the convergence speed of enterprise and service organization still slows down, and government intervention fails to achieve the expected results.

## 5 Discussion

The above research conclusion reveals the core mechanisms and main contradictions in the promotion and application of deep-sea aquaculture. Based on the research conclusion, the following insights can be drawn:

(1) The stability results of the three-party evolutionary game model indicate that relying solely on market mechanisms is insufficient to effectively promote the widespread adoption of deep-sea aquaculture. Government intervention is crucial for the promotion and application of deep-sea aquaculture. However, due to the high cost of deep-sea aquaculture, government intervention is also expensive, and the diminishing marginal effect of subsidy leads to the government eventually withdrawing from the game. Therefore, the promotion and application of deep-sea aquaculture should transition from initial strong government intervention to gradually weakening government intervention. Once the deep-sea aquaculture market and industry are preliminarily established, the promotion and application of deep-sea aquaculture should be fully driven by marketmechanism.
(2) Analysis of initial participation willingness shows that when the initial participation willingness of aquaculture enterprise and service organization is low, the government needs to take measures such as policy promotion and financial subsidy incentives to enhance the participation enthusiasm of both parties. Therefore, the government’s initial participation willingness will increase in the short term. However, as the participation enthusiasm of aquaculture enterprise and service organization increases and the cooperation model between the two parties continues to improve, government intervention will gradually decrease.
(3) Analysis of the impact of risk cost and risk coefficient shows that when the risk cost of deep-sea aquaculture is too high, aquaculture enterprise and service organization cannot form the motivation to participate due to the “loss aversion effect.” Government intervention may lead to the occurrence of “adverse selection,” resulting in government failure. When the proportion of risk borne by aquaculture enterprise and service organization is imbalanced, it will reduce the cooperation efficiency between the two parties and ultimately affect the evolution of the model.
(4) Simulation analysis of cooperation benefit and distribution coefficient reveals the impact of the changing relative importance of “efficiency-equity” on the strategies of aquaculture enterprise and service organization in their cooperation. An increase in cooperation benefit can effectively reduce government intervention cost and enhance the role of market mechanism. However, when the distribution of cooperation benefit loses fairness, the increase in cooperation benefit will generate a reverse driving mechanism, causing a decrease in the enthusiasm of one party to participate.
(5) The phenomenon of double failure of government and market in the analysis of the combined impact of cost and government intervention is primarily due to the government implementing erroneous economic policies that violate market principles. In the context of excessively high cost in deep-sea aquaculture, the focus should be on cost reduction rather than blindly increasing subsidy. This rigid government economic policy not only fails to enhance the efficiency of promoting and applying deep-sea aquaculture but also exacerbates the dependency of aquaculture enterprise and service organization on subsidy, leading to a shift in the motivation for engaging in deep-sea aquaculture from profit-driven to policy arbitrage-driven.

In summary, compared with existing research, the conclusion reached in this study, that “policy guidance should be strengthened and the government should play a leading role in promoting the application of deep-sea aquaculture,” corresponds with the existing research mentioned in the introduction, which confirms the validity of the conclusion reached in this study. Unlike previous studies, this study also obtained other findings through evolutionary game model analysis: (1) Due to the high risk and high cost characteristics of deep-sea aquaculture, government intervention is limited, and the role of market mechanism should be fully utilized. (2) Although profit is an important factor in the promotion and application of deep-sea aquaculture, since deep-sea aquaculture requires the joint efforts of aquaculture enterprise and service organization, it is more important to establish a fair profit distribution and risk-sharing mechanism. (3) The focus of promoting deep-sea aquaculture lies in reducing cost, and blind government intervention policy will not play a positive role. This research proposes concepts such as a “government phased intervention mechanism,” a “risk-sharing and profit-sharing mechanism,” and an “efficiency-equity dynamic relationship” with the aim of providing scientific basis for government policy-making, guiding cooperation between aquaculture enterprise and service organization, and warning of policy failure risk. This study aims to break through the bottlenecks in the promotion of deep-sea aquaculture, increase the supply of high-quality animal protein, empower the construction of blue granaries, and ensure global food security and human nutritional needs.

However, this study still has certain limitations. It only considers the promotion and application of deep-sea aquaculture under the participation of the government, aquaculture enterprise, and service organization, without considering the subsequent circulation, processing, and sales links. Therefore, introducing processing enterprise, logistics enterprise, consumer feedback, and other game subjects or influencing factors will be the direction of future research.

## 6 Policy recommendation

Based on the findings above, the study makes the following policy recommendations:

(1) Increase the initial motivation of deep-sea aquaculture among all participants. As an emerging industry, deep-sea aquaculture is not well understood in most international countries, and its market prospects are still unknown. Therefore, the promotion and application of deep-sea aquaculture should firstly improve the motivation of aquaculture enterprise and service organization. Firstly, policy propaganda on deep-sea aquaculture should be strengthened to enhance social understanding of deep-sea aquaculture through sound top-level design and policy releases, so as to inject strong motivation into the enterprise. Secondly, since aquaculture enterprise and service organization are greatly affected by information asymmetry in their initial decision making, an information and technology exchange platform for deep-sea aquaculture should be established to promote information sharing and technology exchange between aquaculture enterprise and service organization.
(2) Establish a reasonable government intervention system. Government intervention is very important to the promotion and application of deep-sea aquaculture. However, in view of the high-cost characteristics of deep-sea aquaculture, the government should fully respect the rule of the market and take into account its own cost problems, and adopt reasonable intervention measures. First, it is necessary to draw on Norway’s tiered licensing and innovation incentive system to establish an innovative license for deep-sea aquaculture, allowing aquaculture enterprise to apply for excess aquaculture biomass quotas and expand the scale of aquaculture to meet the high-tech and high-risk demands of deep-sea aquaculture. Secondly, in terms of subsidy policies, drawing inspiration from the European Union’s Blue Economy Investment Fund and Blue Carbon Credit Mechanism, a dedicated fund should be established for deep-sea aquaculture. This fund would provide specialized subsidy support for deep-sea aquaculture operations and allow carbon credit revenues to offset a portion of marine area usage fees. Concurrently, the government should establish a subsidy evaluation mechanism to assess subsidy eligibility based on clearly defined subsidy guidelines, standards, and upper limits. Finally, in terms of regulation, cooperation between aquaculture enterprise and service organization supervision should be strengthened, with the latter participating in contract signing as a “notary” and promptly imposing penalties on parties that violate contracts. At the same time, control and tracking of the use of special funds should be strengthened. While maintaining efficiency, the approval process for deep-sea funding support should be strictly enforced to prevent the misuse of national subsidy funds.
(3) Promote cost reduction and improve the risk-sharing and benefit-sharing mechanism. As a high-cost project, the cost of deep-sea aquaculture is an important factor hindering the participating entities. Therefore, in the process of promoting citation, emphasis should be placed on achieving cost reduction. Firstly, the establishment of the deep-sea aquaculture insurance system should be strengthened, and a third-party organization should be involved to assess the risk of deep-sea aquaculture and formulate the insurance policy. At the same time, the government should use its own credibility as a guarantor to reduce the difficulty of the participating parties in obtaining insurance and loans, so as to provide the participating parties with a complete risk guarantee and reduce the risk cost. Secondly, it should actively strengthen the self-research and development of deep-sea aquaculture technology and equipment, promote the transformation of scientific and technological achievements, and improve the legal system of intellectual property rights and patent protection of deep-sea aquaculture, so as to not only reduce the risk cost of service organization, but also enhance the independent innovation ability of deep-sea aquaculture. In terms of risk sharing and benefit sharing, the government should act as a third party to assess the costs and benefits of each party in the process of cooperation in a fair and impartial manner, and determine the criteria for risk sharing and benefit sharing according to the contributions of each party.

## Data Availability

The original contributions presented in the study are included in the article/supplementary material, further inquiries can be directed to the corresponding author.

## References

[1] Kapetsky J. M. Jose A. M. Jenness J. 2013 A global assessment of offshore mariculture potential from a spatial perspective. FAO fisheries and aquaculture technical paper, 549, I

[2] Buck BH Langan R. Aquaculture perspective of multi-use sites in the open ocean: the untapped potential for marine resources in the anthropocene Springer Nature (2017).

[3] Ministry of Agriculture and Rural Development of China (2023) Opinions on accelerating the development of deep-sea aquaculture. Ministry of Agriculture and Rural Affairs of China.

[4] FAO (2022). Fisheries and aquaculture statistics. Global Aquaculture and Fisheries Production 1950–2020 (Fishstatj).

[5] FAO. (2022). The state of world fisheries and aquaculture. Towards blue transformation.

[6] Xu J Han LM Yin W. Research on the ecologicalization efficiency of mariculture industry in China and its influencing factors. Mar Policy. (2022) 137:104935. doi: 10.1016/j.marpol.2021.104935

[7] Merino G Barange M Blanchard JL Harle J Holmes R Allen I. Can marine fisheries and aquaculture meet fish demand from a growing human population in a changing climate? Glob Environ Change. (2012) 22:795–806. doi: 10.1016/j.gloenvcha.2012.03.003

[8] Koričan M Vladimir N Bujas T Vukić M. Comparative analysis of traditional and deep sea aquaculture In: International Symposium on Fisheries and Aquatic Sciences (2023). 74–5.

[9] Zhang Y Li MF Fang XH. Efficiency analysis of China Deep-Sea cage aquaculture based on the SBM–Malmquist model. Fishes. (2023) 8:529. doi: 10.3390/fishes8100529

[10] Zhao YX Zhang JH Qu DP Yang YY Wu WG Sun K. Benthic environmental impact of deep sea cage and traditional cage fish mariculture in Yellow Sea, China. Aquac Res. (2021) 52:5022–33. doi: 10.1111/are.15374

[11] Yin W Han LM Xu JJ Guo AH. Discussion on key issues in the development of China’s deep-sea aquaculture industry chain: a case study of salmon farming in the Yellow Sea cold water mass. China Fish Econ. (2023) 41:25–33.

[12] Yin W Yu HJ Qiu RS Han LM. China’s food and nutrition security in the context of land-sea integration. Resour Sci. (2022) 44:674–86.

[13] Yu HJ Niu M Han LM. Development strategies for China’s “blue granary” and industrial chain restructuring. Agric Econ Issues. (2019) 11:72–81. doi: 10.13246/j.cnki.iae.2019.11.007

[14] Froehlich HE Smith A Gentry RR Halpern BS. Offshore aquaculture: I know it when I see it. Front Mar Sci. (2017) 4:154. doi: 10.3389/fmars.2017.00154

[15] Greaker M Vormedal I Rosendal K. Environmental policy and innovation in Norwegian fish farming: resolving the sea lice problem. Mar Policy. (2020) 117:103942. doi: 10.1016/j.marpol.2020.103942

[16] Afewerki S Osmundsen T Olsen MS Størkersen KV Misund A Thorvaldsen T. Innovation policy in the Norwegian aquaculture industry: reshaping aquaculture production innovation networks. Mar Policy. (2023) 152:105624. doi: 10.1016/j.marpol.2023.105624

[17] Føre HM Thorvaldsen T Osmundsen TC Moe Føre H Asche F Tveterås R. Technological innovations promoting sustainable salmon (*Salmo salar*) aquaculture in Norway. Aquac Rep. (2022) 24:101115. doi: 10.1016/j.aqrep.2022.101115

[18] Huan XM Shan JZ Han LM Song HM. Research on the efficacy and effect assessment of deep-sea aquaculture policies in China: quantitative analysis of policy texts based on the period 2004–2022. Mar Policy. (2024) 160:105963. doi: 10.1016/j.marpol.2023.105963

[19] Wu KK Li QS Huang HP Chen KL. Deep-sea aquaculture progress and development measures in China. Marine Dev Manag. (2022) 39:11–8. doi: 10.20016/j.cnki.hykfygl.20221014.001

[20] Zhang ZZ Liu YY. The status quo, issues and foreign experiences of Deep Sea aquaculture equipment of China. Int Conf Soc Sci. (2018):373–7.

[21] Lin M. Development of large-scale deep-sea aquaculture: problems, models and realisation paths. Manag World. (2022) 38:39–60. doi: 10.19744/j.cnki.11-1235/f.2022.0177

[22] Gao S. Development direction and suggestion of deep-sea aquaculture equipment and facilities. China Water Transport. (2023) 23:14–5.

[23] Liu HM Bi WJ Wu P. Current situation and suggestions on the development of deep-sea aquaculture in Weihai city. Aquaculture. (2023) 44:61–62+68.

[24] Lin JM. Current situation and suggestions for the development of equipment-based deep-sea aquaculture in Fujian Province. Fish Modernis. (2024) 51:11–8.

[25] Chen CP Liu HF Huang Y Yang J Liang XY Zhang CB. Numerical simulation of mechanical characteristics of a metal net for deep-sea aquaculture. J Ocean Univ China. (2019) 18:1273–81. doi: 10.1007/s11802-019-4079-z

[26] Tao YW Zhu RQ Gu JY Li ZY Zhang ZY Xu XS. Experimental and numerical investigation of the hydrodynamic response of an aquaculture vessel. Ocean Eng. (2023) 279:114505. doi: 10.1016/j.oceaneng.2023.114505

[27] Hou HM Dong GH Xu TJ. Analysis of probabilistic fatigue damage of mooring system for offshore fish cage considering long-term stochastic wave conditions. Ships Offshore Struct. (2020) 17:398–409. doi: 10.1080/17445302.2020.1834752

[28] Li G Yao ZD Hu Y Lian AJ Yuan TP Pang GL. Deep learning-based fish detection using above-water infrared camera for deep-sea aquaculture: a comparison study. Sensors. (2024) 24:2430. doi: 10.3390/s24082430, PMID: 38676049 PMC11054504

[29] Wang PH Chen ZG Feng YK. Many-objective optimization for a deep-sea aquaculture vessel based on an improved RBF neural network surrogate model. J Mar Sci Technol. (2021) 26:582–605. doi: 10.1007/s00773-020-00756-z

[30] Yuan XN Miao QM Zou CF Wang CY. Numerical study on aquaculture environment in deep-sea cage with waves. ISOPE Int Ocean Polar Engi Conf. (2022)

[31] Zhang QF Wang LK Chen MJ. Construction technology for large-scale frame-type deep-sea aquaculture cages. Marine Dev Manag. (2024) 41:83–90. doi: 10.20016/j.cnki.hykfygl.20240923.001

[32] Chen SX Su YQ Hong WS. Aquaculture of the large yellow croaker. Aquac China. (2018):297–308. doi: 10.1002/9781119120759.ch3_10

[33] Fan F Zheng JL Liu H Cui MC. Assessment of the carbon footprint of large yellow croaker farming on the aquaculture vessel in deep sea in China. J Mar Sci Eng. (2024) 12:693. doi: 10.3390/jmse12050693

[34] Li TJ Guo DK Shen Y Bao JJ Jin L. Comparative analysis of bacterial communities in the sediment and seawater environments from marine large yellow croaker cages (Zhejiang coast, China). Front Mar Sci. (2022) 9:963242. doi: 10.3389/fmars.2022.963242

[35] Li YY Zhen XW Zhu YS Huang Y Zhang LX Li HX. Conceptual design and structural performance analysis of an innovative deep-sea aquaculture platform. J Mar Sci Eng. (2024) 12:1058. doi: 10.3390/jmse12071058

[36] Føre M Alver MO Frank K Alfredsen JA. Advanced technology in aquaculture–smart feeding in marine fish farms. Cham: Springer (2023).

[37] Yuan XN Wang CY Miao QM Zou CF. Investigation on quantitative evaluation method for feed diffusion effect in deep-sea aquaculture cages. Ocean Eng. (2024) 295:116759. doi: 10.1016/j.oceaneng.2024.116759

[38] Traulsen A Glynatsi NE. The future of theoretical evolutionary game theory. Philos Trans R Soc B. (2023) 378:20210508. doi: 10.1098/rstb.2021.0508, PMID: 36934760 PMC10024985

[39] Gao XL Lu WW Hu J Zhang XL. Current status and challenges of deep-sea aquaculture industrialization in China: a study based on Liaoning, Shandong, Guangdong, and Hainan. Marine Econ. (2025) 15:11–23. doi: 10.19426/j.cnki.cn12-1424/p.2025.02.002

[40] Ning J Xiong L. Analysis of the dynamic evolution process of the digital transformation of renewable energy enterprises based on the cooperative and evolutionary game model. Energy. (2024) 288:129758. doi: 10.1016/j.energy.2023.129758

[41] Wang EK Chen CM Yiu SM Hassan MM Alrubaianm M Fortino G. Incentive evolutionary game model for opportunistic social networks. Futur Gener Comput Syst. (2020) 102:14–29.

[42] Zhu LL Rong JM Zhang SY. Evolutionary game theory and simulation analysis of three-party interaction in drug safety and quality supervision under government reward and punishment mechanisms. Chin J Manage Sci. (2021) 29:55–67. doi: 10.16381/j.cnki.issn1003-207x.2019.0481

